# Long-Lasting Decrease of the Acquisition of *Enterococcus faecium* and Gram-Negative Bacteria Producing Extended Spectrum Beta-Lactamase (ESBL) by Transient Application of Probiotics

**DOI:** 10.3390/ijerph17176100

**Published:** 2020-08-21

**Authors:** Stefan Borgmann, Beate Rieß, David Meintrup, Ingo Klare, Guido Werner

**Affiliations:** 1 Hospital of Ingolstadt, Department of Infectious Diseases and Infection Control, D-85049 Ingolstadt, Germany; beate.riess@klinikum-ingolstadt.de; 2 Technische Hochschule Ingolstadt, Faculty of Engineering and Management, D-85049 Ingolstadt, Germany; David.Meintrup@thi.de; 3 National Reference Centre for Staphylococci and Enterococci, Robert Koch Institute, Wernigerode Branch, D-38855 Wernigerode, Germany; ingo.klare@gmx.de (I.K.); wernerg@rki.de (G.W.)

**Keywords:** colonic bacteria, *Enterococcus faecium*, vancomycin-resistant *Enterococcus faecium*, vancomycin-susceptible *E. faecium*, *Staphylococcus aureus*, beta-lactamase, probiotics, health care co-workers, electric current, transistor, condenser, electrical current hypothesis of bacterial transmission

## Abstract

Previously it was shown that application of probiotics stopped the acquisition of vancomycin-resistant *Enterococcus faecium* (VRE) by patients in an early rehabilitation ward. Once the application of probiotics ended, we examined whether acquisition of VRE reoccurred. Furthermore, we examined whether probiotics altered prevalence of vancomycin-susceptible *E. faecium* (VSE) and Gram-negative bacteria, which produce extended spectrum beta-lactamase (ESBL). Although probiotic application ceased in April 2018, VRE-colonized patients rarely presented on that ward until 2019. Probiotic treatment also resulted in a decreased number of patients with VSE and ESBL. While decreased incidence of VRE occurred immediately, decreased VSE and ESBL numbers occurred months later. A probiotic-mediated decrease of VSE and ESBL incidence cannot be explained when assuming bacterial transmission exclusively as a linear cause and effect event. The decrease is better understood by considering bacterial transmissions to be stochastic events, which depend on various driving forces similar to an electric current. We hypothesize that VRE, VSE and ESBL uptake by patients and by staff members mutually reinforced each other, leading staff members to form a bacterial reservoir, similar to a condenser that stores electrical energy. Probiotic treatment then inhibited regeneration of that store, resulting in a breakdown of the driving force.

## 1. Introduction

Antibiotic resistance is a global burden complicating treatment of bacterial infections. It is estimated that by 2050, up to 300 million premature deaths may be attributable to infections with multi-resistant bacteria [1]. An increasing number of Gram-negative bacteria, mainly enterobacteriaceae, are able to produce enzymes that destroy penicillin and cephalosporin antibiotics. Bacteria producing enzymes that inactivate third generation cephalosporin antibiotics are referred to as extended spectrum beta-lactamase (ESBL) producers if the genes encoding these enzymes are located on plasmids, which facilitate transmission to other bacteria. Apart from multi-resistant Gram-negative bacteria, vancomycin-resistant *Enterococcus faecium* (VRE) was indexed by the World Health Organization (WHO) as a pathogen for which new treatments are urgently needed [2]. Except for the availability of novel antibiotics, rational use of medications and the application of probiotics might be another way to prevent colonization with multi-resistant pathogens [3].

Probiotics are living microorganisms capable of mediating beneficial effects to a host when applied in appropriate amounts [4]. Frequently administered probiotics include various species of lactobacilli (e.g., various strains of *Lactobacillus rhamnosus*, *L. reuteri*), *Bifidobacterium spp.*, and *Saccharomyces boulardii*. *S. boulardii* is a yeast that is genetically close to *S. cerevisiae* but shows a higher tolerance to acid pH than *S. cerevisiae* [4]. In the gut, *S. boulardii* mediates various beneficial effects to the host. Firstly, pathogen exclusion most likely results from pathogens binding to the yeast cell. Secondly, the secretion of proteins prompts antimicrobial action to pathogens. Thirdly, immune modulation decreases the level of pro-inflammatory cytokines. Finally, trophic effects maintain a functional boarder to the gut lumen [4,5,6,7].

*Escherichia coli* Nissle is a probiotic bacterium originally isolated during World War I from the stool of a German soldier who, unlike his army comrades, did not develop diarrhea during a stay in a *Shigella*-contaminated region [8]. In addition to the treatment of diarrhea and other gastrointestinal diseases, *E. coli* Nissle is applied to maintain remission of ulcerative colitis [9]. *E. coli* Nissle kills and inhibits growth of pathogenic bacteria and yeasts, augments intestinal colonization resistance and favorably modulates host cell signaling [8,9].

The primary aim of treatment on an early rehabilitation ward is to improve functional outcome following acute treatment in a hospital. Infections are a common complication for patients in these facilities. Longer hospital stays likely cause patients to experience colonization with multi-resistant bacteria, which are associated with poorer outcomes [10].

Due to increasing prevalence, VRE are a growing problem in southern Germany [11]. Recently, we reported that the treatment of patients receiving antibiotics with *S. boulardii* and *E. coli* Nissle stopped a long-lasting outbreak of VRE on an early rehabilitation ward [12]. Following discontinuation of probiotics on that ward, patients were later examined to determine the rate at which new VRE infections were occurring. Furthermore, we examined the effect of probiotics on the acquisition of vancomycin-susceptible *E. faecium* and Gram-negative bacteria producing ESBL. Based on the results of these analyses, we present a new transmission model for potentially pathogenic colonic bacteria in a high incidence setting.

## 2. Patients and Methods

The Ingolstadt Hospital is a tertiary care hospital with close to 1200 beds. Ingolstadt is located in the center of the federal German state of Bavaria (South-East Germany). The early rehabilitation ward of the Ingolstadt Hospital (ERWIN) is comprised of 22 beds. Additional features of the ward have been previously reported [13]. Patients treated on the ward primarily suffer from neurological deficits, resulting mostly from stroke and trauma sequalae (≥2 injuries).

In winter 2015, ERWIN closed for several weeks due to an outbreak of carbapenem-resistant *Klebsiella pneumoniae* (CKP) [13]. The ward later reopened (February 2015), following an intense cleaning and disinfection process.

Patients in the Ingolstadt Hospital were screened to identify colonization/infection with pathogenic and/or multi-resistant bacteria in accordance with the recommendations of the Commission on Hospital Hygiene and Infection Protection (KRINKO) at the Robert Koch Institute (RKI). Screening patients for colonization/infection with methicillin-resistant *S. aureus* (MRSA) and multi-resistant Gram-negative bacteria (MRGN), among them ESBL, occurred when patients exhibited certain risk factors as defined by the KRINKO [14,15]. To screen for ESBL, rectal swabs were taken and, for MRSA, nasal swabs. Wounds were also screened for colonization with MRSA. Beyond the KRINKO recommendations, nasal screening of patients with risk factors was repeated once per week.

In contrast to the other wards in the hospital, screening efforts on ERWIN were more extensive. Beginning in March 2015, each patient was screened for MRSA and ESBL upon admission and then once per week. Screening for MRGN and MRSA was initiated after reopening the ward in February 2015. Between May 2014 and November 2016 rectal swabs were also taken upon admission and once per week to assess colonization with vancomycin resistant *Enterococcus faecium* [12]. Between 2011 and 2019 in total 2773 patients were treated on ERWIN (median = 294 patients per year; range = 251–405). According to the KRINKO, 3- and 4-MRGN are Gram-negative bacteria (enterobacteria and some nonfermenter species), that exhibit resistance to three or four of the following antibiotics or antibiotic groups: Piperacillin, cephalosporines belonging to group 3 or 4, quinolones, and carbapenems [15]. In accordance with the KRINKO guidelines, patients colonized/infected with 4-MRGN were contact-isolated on each ward of the hospital, while patients with 3-MRGN underwent contact isolation only on the intensive care units (ICUs) and on the hematology-oncology wards. Patients colonized/infected with VRE resided in a single room or underwent cohort isolation with a sex-matched VRE-colonized roommate. The same held true for patients colonized/infected with MRSA.

Staff and visitors contacting isolated patients wore gloves and coats and were instructed to carefully maintain basic hygiene measures, primarily hand disinfection.

**Figure 1:**
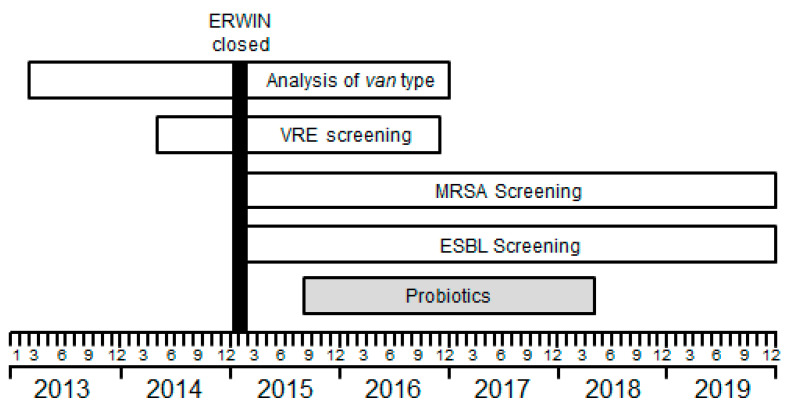
Overview of infection control measures performed on early rehabilitation ward of Ingolstadt hospital (ERWIN) between January 2013 and December 2019. Rectal swabs were taken from each patient treated on ERWIN at admission and once per week between May 2014 and November 2016 to identify colonization with vancomycin-resistant *Enterococcus faecium* (VRE). From 2013–2016, the type of the gene (*van*A, *van*B, etc.) that mediated vancomycin resistance of VRE was systematically examined. ERWIN was temporarily closed during January and February 2015 for deep cleaning and disinfection of the ward following an outbreak of carbapenem-resistant *Klebsiella pneumonia*. Upon reopening the ward, each patient was screened at admission and once per week for colonization with extended spectrum beta-lactamase (ESBL)-producing Gram-negative bacteria and methicillin-resistant *Staphylococcus aureus* (MRSA). From 2010 to 2012, no additional infection control measures were performed on ERWIN.

From September 2015 to April 2018, patients on ERWIN received two probiotic drugs when treated with antibiotics. Probiotics were *Saccharomyces boulardii* 375 mg/hard capsule, (Eubiol; CNP-Pharma GmbH, Fürstenzell, Germany) and *E. coli* Nissle 2.5–25 × 10^9^ bacteria/capsule (Mutaflor; Ardeypharm GmbH, Herdecke, Germany). *S. boulardii* was given once a day (1-0-0) and *E. coli* Nissle twice a day (1-0-1). Probiotics were applied during antibiotic treatment and for two additional days. The chronological process of infection control measures is summarized in Figure 1.

As reported earlier, the Ingolstadt Hospital’s laboratory performed microbiological diagnostics [13,16]. In brief, bacteria from screening swabs were grown on culture plates containing blood or selective agar, whereas bacteria in clinical samples were cultured on standard media. VRE were cultured on Brilliance VRE agar (Oxoid, Wesel, Germany), MRSA on ID-Agar (BioMerieux, Nürtingen, Germany), and multi-resistant Gram-negative bacteria (MRE) were grown on Gram-negative agar plates divided into halves (Oxoid, Wesel, Germany), containing either Brilliance ESBL agar or Brilliance CRE agar for identification of carbapenem-resistant Gram-negative bacteria. Species identification and antibiotic susceptibility was examined with the Vitek 2 compact (BioMerieux, Nürtingen, Germany). For identification of VRE, the GP ID and the AST-P586 card were used. Vancomycin resistance was confirmed by determining minimal inhibitory concentration (MIC) with the vancomycin Etest (BioMerieux, Nürtingen, Germany). MIC ≤ 4 mg/L was considered as susceptible [16].

Characteristic colonies grown on MRSA agar were initially examined by using the Pastorex Staph Plus kit (BioRad, Munich, Germany) and PBP2a Culture Colony Test (Alere, Cologne, Germany), and were subsequently analyzed in the Vitek 2 with the GP ID and the AST-P580 card.

Suspicious colonies grown on ESBL and/or CRE agar were cultured on MacConkey agar (BioMerieux, Nürtingen, Germany) and subsequently examined in Vitek 2 using GN ID card and Enterobacteriaceae AST-N233 or *Pseudomonas aeruginosa* AST-N248 card. Presence of ESBL was identified by performing the double synergy test using cephalosporine containing discs (MAST Diagnostica, Rheinfeld, Germany) and Etest stripes (BioMerieux, Nürtingen, Germany) with and without clavulanic acid.

In addition to screening cultures, VRE, MRSA and ESBL were also isolated from clinical samples (wounds, urine, etc.), while methicillin-susceptible *S. aureus* (MSSA) and vancomycin-susceptible *E. faecium* (VSE) were exclusively obtained from clinical samples. Samples for routine diagnostics were cultured for 48 h on Schaedler agar +5% sheep blood (BioMerieux, Nürtingen, Germany), blood (BioMerieux, Nürtingen, Germany), and MacConkey agar (BioMerieux, Nürtingen, Germany) and urine was cultured on blood (BioMerieux, Nürtingen, Germany) and MacConkey agar (BioMerieux, Nürtingen, Germany).

The results of the microbiological analyses were sent to the computer program Hybase (EpiNetAG, Bochum, Germany). This program allows the identification of patients colonized/infected with a particular bacterium. In our setting, *S. aureus* and MRSA, as well as *E. faecium* and VRE, are encoded as two different species, respectively. The same holds true for Gram-negative bacteria and their corresponding ESBL variants. This allows for fast and easy access to the name of VRE-colonized patients and the date of bacterial isolation during a certain period, e.g., 2011–2019 by using the Hybase program. Negative screening results of VRE-colonized patients were obtained from the laboratory software LabCentre l.i.c. laboratory software (i-SOLUTION Health GmbH, Mannheim, Germany). Number of patient days were received from the computer program industry solutions healthcare module (SAP/IS-H; Siemens, Munich, Germany), adapted to the requirements of the hospital. From March 2013 to December 2016 VRE were systematically examined by polymerase chain reaction to discover genotype mediating vancomycin resistance at the Robert-Koch-Institute (RKI), as described previously [12]. Since January 2017 only VRE isolated from blood cultures were analyzed at the RKI.

## 3. Results

As reported earlier, the application of two probiotics to patients on ERWIN who were being treated with antibiotics inhibited the spread of vancomycin-resistant *E. faecium* (VRE). In the present analysis, we examined whether application of probiotics also reduced detection rates of other unwanted bowel bacteria. In addition, the number of VRE-colonized patients was evaluated to examine how the number of VRE was impacted once application of probiotics ceased in April 2018. The absolute number of patients on ERWIN found to be colonized by these bacteria is summarized in Table 1, the corresponding number of patients on the other wards in Table 2.

**Figure 2:**
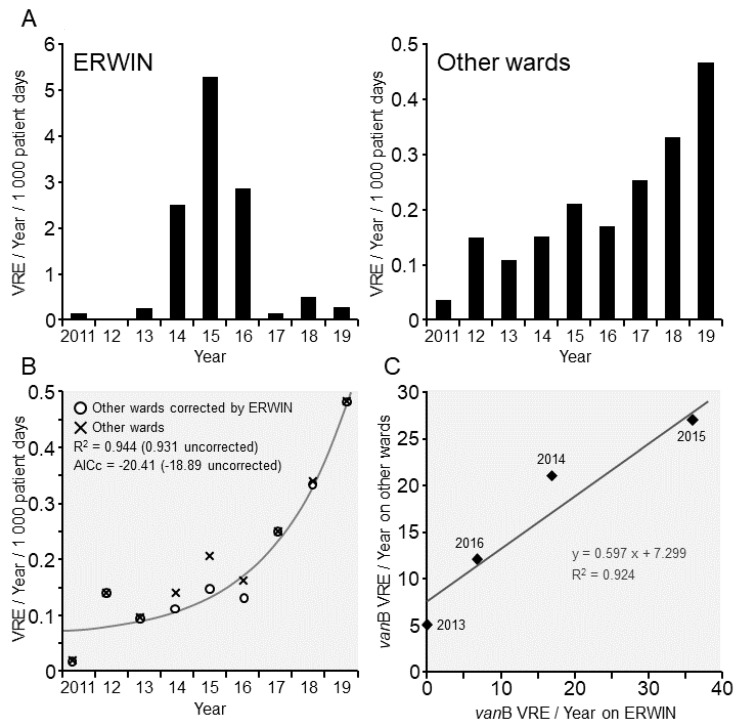
Vancomycin resistant *E. faecium* (VRE) at Ingolstadt Hospital (2011–2019). (**A**) Number of patients colonized with VRE per 1000 patient days per year. Data were obtained from screening and clinical samples. **Left panel**: Patients on ERWIN. **Right panel**: Patients on other wards. (**B**) Exponential growth model fitted to corrected VRE rates on other wards. Correction was performed proportionally to rates on ERWIN. The model shows a very good fit, better than the fit for uncorrected data. (**C**) Number of patients colonized with *van*B VRE. In the figure, only data from 2013–2016 are shown because within this period identical conditions were present (systematic *van*-type analysis and VRE screening on ERWIN). AICc = Corrected Akaike information criterion.

As previously shown [12], application of probiotics markedly dropped the number of VRE-colonized patients on ERWIN, and the number remained low until 2019 (Figure 2A). In contrast to ERWIN on the other wards, the number of VRE-colonized patients continuously increased from 2016 and, in 2019, the rate was within a similar range to that on ERWIN (0.46 (other wards) vs. 0.29 colonized patients per 1000 patient days (ERWIN)). In 2015, the colonization rate on ERWIN had been much higher than on the other wards (5.92 vs. 0.21 colonized patients per 1000 patient days). The increase of the VRE-colonization rate on the other wards was due to two causes. First, there is an underlying exponential growth of VRE cases. Second, some cases on the other wards were triggered by the outbreak on ERWIN. These statements were verified by fitting an exponential growth model to the corrected VRE cases on the other wards (Figure 2B). The correction was performed by subtracting a number of cases proportional to the cases on ERWIN. We found the optimal proportionality factor for this transfer effect to be 0.9%. The exponential model fits the corrected data very well and better than the uncorrected data, as was shown with two different model evaluation criteria, R^2^ (based on sums of squared errors), and AICc (based on the likelihood function). Finally, we observed a very high correlation between *van*B VRE-colonized patients on ERWIN and on the other wards (Figure 2C), which is another indicator supporting the connection between the two colonization developments.

**Figure 3:**
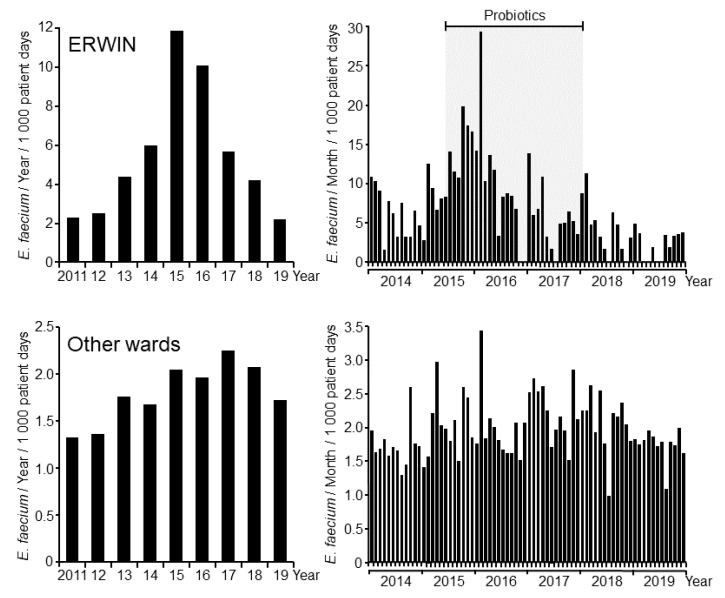
Number of patients colonized per 1000 patient days with vancomycin-susceptible *E. faecium* at Ingolstadt Hospital per year (**left panels**) and per month (**right panels**). Data were obtained from clinical samples only. **Upper panels**: Patients on ERWIN. **Lower panels**: Patients on other wards.

As described earlier, probiotic application immediately reduced VRE colonization rates [12]. In contrast, the detection rate of VSE within the first months following probiotic treatment was higher than in the previous months. However, the number of VSE-colonized patients later markedly diminished on ERWIN (Figure 3). Due to the apparent differing chronological courses of VSE colonization rates on ERWIN and on the other wards, is reasonable to conclude that reduced detection rates on ERWIN had a local cause and occurred independently from what developed outside that ward.

**Figure 4:**
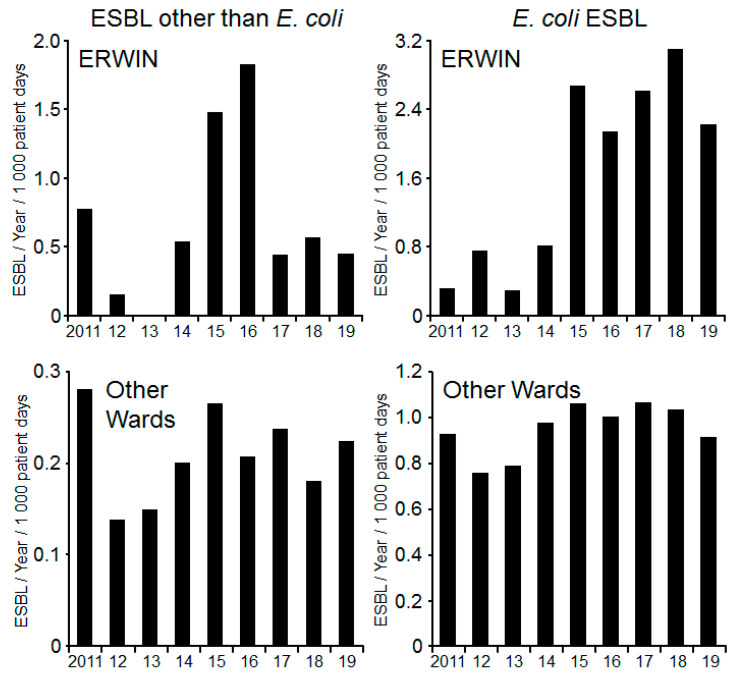
Number of patients colonized per 1000 patient days with extended spectrum beta-lactamase (ESBL)-producing Gram-negative bacteria at the Ingolstadt Hospital per year. Data were obtained from screening and clinical samples. **Left panels**: Other ESBL-producing species than *Escherichia coli*. **Right panels**: ESBL-producing *E. coli*. **Upper panels**: Patients on ERWIN. **Lower panels**: Patients on other wards.

Between 2011 and 2014 on ERWIN 0 to 10 patients were identified as being colonized with ESBL-producing Gram-negative bacteria. In 2015 this number tripled, as each patient on the ward was screened once per week for colonization with multi-resistant Gram-negative bacteria following an outbreak of multi-resistant *Klebsiella pneumoniae*. The application of probiotics per se did not reduce the frequency of ESBL colonized patients. However, when separating ESBL-producing bacteria into *E. coli* ESBL and other ESBL producer (EOEC), it becomes evident that the rate of patients colonized with EOEC was markedly reduced following application of probiotics (2017–2019) compared to the rate observed before initiation of probiotic treatment (2015–2016). On the other wards, this development was not observed, suggesting a ward-specific cause of diminished colonization rate, e.g., application of probiotics. However, on ERWIN, the number of patients colonized with *E. coli* ESBL remained high, even after initiating probiotic treatment (Figure 4).

**Figure 5:**
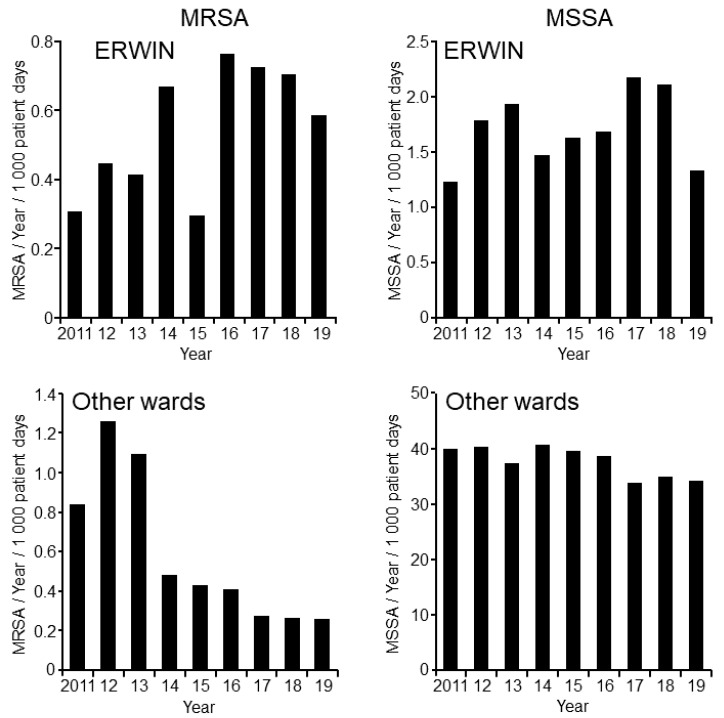
Number of patients colonized per 1000 patient days with methicillin resistant *S. aureus* (MRSA) (**Left panels**) and methicillin-susceptible *S. aureus* (MSSA) (**Right panels**) at the Ingolstadt Hospital per year. Data for MRSA were obtained from screening and clinical samples, while data for MSSA were obtained from clinical samples only. **Upper panels**: Patients on ERWIN. **Lower panels**: Patients on other wards.

Finally, the number of *S. aureus* (MRSA and MSSA) was examined to assess whether decreased detection rates of VSE and ESBL resulted from probiotic treatment or from a different unknown source. As shown in Figure 5, the number on patients on ERWIN colonized with *S. aureus* (MSSA and MRSA) did not decrease after initiating probiotic treatment. Furthermore, the chronological sequence of the *S. aureus* detection rates on ERWIN was generally within the range of that on other wards. This observation indicates that, above all else, oral application of probiotics to patients being treated with antibiotics inhibited colonization with potentially pathogenic colonic bacteria.

While patients on ERWIN were screened weekly for VRE, ESBL producer and MRSA, the number of VSE- and MSSA-colonized patients was estimated from analysis of clinical samples. To assess the percentage of patients infected by the aforementioned bacteria, the number of patients exhibiting those bacteria in blood cultures was determined (Table 3). No ESBL-producing bacteria were found in blood cultures. While VRE, VSE and MRSA were only sporadically isolated, up to six MSSA were isolated per year from blood cultures. On the other hand, MRSA colonization more frequently led to systemic infections when compared to colonization with VRE and VSE. Therefore, colonization with *S. aureus* resulted in a systemic infection relatively often while this was not the case when colonized with *E. faecium* or ESBL producer.

## 4. Discussion

This monocentric retrospective study examines the application of two probiotics that resulted in a reduced number of patients colonized with potentially pathogenic bacteria on an early rehabilitation ward. In September 2015 on ERWIN, probiotic treatment was initiated to limit the spread of VRE. The present analysis reveals that apart from VRE, the number of patients colonized with EOEC also dropped, presumably as an outcome of probiotic treatment. Furthermore, the number of patients colonized or infected with VSE decreased.

Compared to patients on other wards of the hospital, patients on ERWIN were more frequently colonized with unwanted bowel bacteria. By contrast, colonization rates of *S. aureus* (MSSA and MRSA) were similar in both patient groups. This finding indicates that, in particular, disturbances of the gut microbiome were a matter of concern on ERWIN. Patients on ERWIN often suffer from dysphagia due to stroke and must learn to swallow again. During this process, patients often spit up food or liquids, especially when fed nonadequate food by their relatives. For treatment of the ensuing pneumonia, patients frequently receive broad-spectrum antibiotics that also inhibit the growth of anaerobic bacteria [12]. As a result, the gut microbiome is disturbed, thus facilitating colonization with potentially harmful and/or multi-resistant colonic bacteria. Evidently, consumption of the probiotics prevented colonization with unfavorable bacteria.

*E. faecium* was rarely isolated from infections in humans before the 1990s. Since then, the bacteria have been isolated from patients in hospitals with increasing frequency. Originally, this species was susceptible to ampicillin and many *E. faecium* isolated from individuals in the community remained susceptible [17,18]. With increasing frequency, patients in hospitals have become colonized with hospital-associated, ampicillin-resistant *E. faecium,* replacing the ampicillin-susceptible human commensal *E. faecium* [19,20]. Accordingly, only 18 of 339 (5.3%) VSE isolated between 2011 and 2019 from patients on ERWIN were ampicillin-susceptible, supporting the idea that the vast majority of VSE had been acquired during stationary care (Table 1). As previously shown, the number of VRE-colonized patients decreased immediately following initiation of probiotic treatment [12]. By contrast, within the first months after the start of probiotic treatment, the number of VSE positive patients was higher than ever before.

There are two possible explanations for this surprising finding. The first is that the colonization of patients with VSE occurred before probiotic treatment was initiated. However, this idea implies that colonization with VSE was noticed much later than with VRE. In contrast to VSE, patients on ERWIN were screened once per week for VRE. The cultivation of VRE was determined by using a selective medium that inhibits growth of competing bacteria, therefore, allowing detection of low bacterial density. By contrast, carriage of VSE would have been obvious if the bacterial number had been high enough to be detected in clinical samples (mostly urine). The second explanation is that there existed a “bacterial reservoir” maintaining acquisition of VSE by the patients. Although we know of no analyses demonstrating that medical personnel contributed to spread of VRE and VSE, we hypothesize that members of the staff functioned as that reservoir, contributing to acquisition of VSE by patients. Concurrently, staff members acquired VSE from patients, thus, perpetuating a vicious cycle of VSE transmission between patients and staff members. This occurred despite the implementation of infection control measures. Eventually, this vicious cycle must have broken down because the number of patients with VSE decreased due to the application of probiotics.

Once probiotic treatment started, the number of EOEC declined, whereas the colonization frequency with ESBL-*E. coli* remained unchanged. This observation exists in contrast to previously reported frequencies of ESBL-*E. coli* in medical facilities [21]. Nevertheless, it is generally recognized that the bacteria are mainly acquired in the community, independent of antibiotic uptake [22]. Consequently, application of probiotics did not facilitate the loss of ESBL-*E. coli* [23]. Furthermore, there was a substantial percentage of ESBL-*E. coli* detected among healthy German community residents [24], leading to the conclusion that contact precautions are not necessary to limit spread of ESBL-*E. coli* in the majority of clinical settings [25]. When comparing transmission patterns of ESBL-*E. coli* and EOEC the analyses are mostly limited to ESBL-*Klebsiella pneumoniae* because these bacteria are most frequently isolated EOEC.

In healthcare settings, close proximity interactions support transmission of ESBL-*K. pneumoniae* but not transmission of ESBL-*E. coli* [26]. Accordingly, in a hospital setting acquisition of ESBL-*E. coli* was lower than that of ESBL-*K. pneumoniae*. In contrast to ESBL-*K. pneumoniae*, net transmission rate of ESBL-*E. coli* was higher in household settings than in the hospital setting, presumably due to longer exposure time [27]. As observed by others, the impact of nosocomial transmission on the prevalence of EOEC is higher than that on the prevalence of ESBL-*E. coli* [22,23,24,25,26,27]. Consequently, probiotic treatment should have a higher impact on the transmission of EOEC than on the transmission of ESBL-*E. coli,* as observed in the present study.

Interestingly, from 2016 to 2017, the number of EOEC declined more than from 2015 to 2016. This finding might be explained by a vicious cycle of reciprocity between patients and staff members. However, since the beneficial effect of probiotic treatment on colonization rates in staff members of EOEC was obvious much later than that on VSE, the duration of EOEC carriage should have been much longer than that of VSE. To date, data pertaining to the duration of the colonization with EOEC are rare, since the vast majority of ESBL producer are *E. coli* [23,28].

In a large outbreak within a German university hospital with pan-resistant *K. pneumoniae*, the majority of patients showed no signs of the bacteria after 6 months, although some patients remained colonized for more than 3 years, supporting the idea of long-lasting colonization with EOEC [29]. However, pan-resistant *K. pneumoniae* differed from EOEC since the bacteria were KPC-2 positive and it is not clear to which degree carriage duration of both entities were comparable.

A limitation of our analysis is that staff members were not examined for colonization with VRE, VSE, and EOEC. On the other hand, even when searching extensively for outbreak bacteria, there was no proof that staff members had been colonized [30,31]. Due to lack of antibiotic treatment, the microbiome of staff members usually is not compromised and, consequently, the extent of the colonization is much lower, possibly so low that colonization will not be detected when taking rectal swabs. Nevertheless, this low-grade colonization seems to contribute to the spread of the bacteria, as suggested by the present data. When applying probiotics to patients treated with antibiotics, the reproduction rate of resistant bacteria decreases to the extent that the bacterial uptake for staff members breaks down, driving the degree of colonization to such a point that the vicious cycle of give and take collapses.

**Figure 6:**
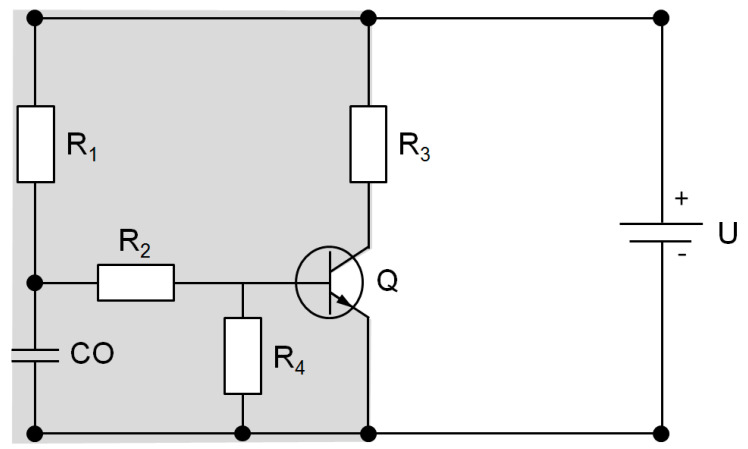
Circuit diagram as a model of *van*B VRE transmission on the early rehabilitation ward (**grey area**, **left side**) of Ingolstadt Hospital (ERWIN) and other wards of the hospital (**white area**, **right side**). R_1_ = Resistor representing infection control measures on ERWIN. R_2_ = Resistor resulting from probiotic treatment. R_3_ = Resistor composed of infection control measures on the other wards of the hospital. R_4_ = Resistor necessary due to technical reasons. Q = Transistor enhancing spread of *van*B VRE on the other wards depending on the VRE situation on ERWIN. CO = Condenser representing VRE load of staff members. U = Driving force of VRE transmission in the hospital.

The authors realize that this idea is not in line with the current model assuming that bacterial transmission is a linear cause-and-effect phenomenon. As shown above, it is necessary to regard bacterial transmission as a set of stochastic events, depending on various driving forces similar to that of an electric circuit. In the model presented in the present study, maintenance of bacterial transmission following introduction of probiotics might be comparable to the functioning of a capacitor (condenser), which maintains an electrical circuit after a driving force has broken down. Such a capacitor is capable of storing electrical energy, which can eventually be released once the circuit breaks down. This theory corresponds to the observation that between 2013 and 2015, a high number of *van*B-colonized patients were accompanied by an increased number of *van*B-colonized patients on the other wards. However, when the number of colonized patients decreased in 2016, the number of colonized patients also decreased on the other wards (Figure 2C). Typically, patients are treated on other wards before admission to ERWIN. Therefore, it is not probable that the transient increase of *van*B VRE incidence was caused by transmissions from patients on ERWIN to patients on the other wards. Although the number of patients identified to be colonized with *van*B VRE on ERWIN and on the other wards was in a similar range (Figure 2), on the other wards the real number of *van*B VRE probably was much higher than that on ERWIN. Due to the fact that patients on ERWIN were actively screened for VRE, the majority of *van*B-colonized patients should have been identified. On the other wards, patients were not screened for VRE colonization. Therefore, only a small portion of colonized patients were identified and the actual number of *van*B VRE-colonized patients should have been much higher than the number of patients identified to be colonized. Taking this into account and the fact that the incidences on ERWIN and the other wards were synchronized, a small number of *van*B-colonized patients most likely steered a large number of colonization on the other wards. This phenomenon relates to how a transistor functions. The transistor is an electronic component that harmonizes the fluxes of an electric circuit with a high current to the fluxes of a circuit with a lower current, thereby amplifying the signal of the low current circuit. Interestingly, the transistor exhibits a threshold voltage that must be reached within the circuit of the lower current to allow current flow in the higher current circuit. We hypothesize that application of probiotics mediated an effect similar to that of a built-in resistor, decreasing the voltage below the threshold of the transistor (Figure 6). However, we cannot exclude that the parallel increase and decrease of *van*B isolation frequencies on ERWIN and the other wards was caused by an undetected confounding factor. Nevertheless, even if our model does not fully capture the complexity of bacterial transmission, it might be suited to show that the net effect of various driving forces determinates whether pathogen transmission happens or not.

Our study has further limitations. The number of VRE, ESBL and MRSA were obtained from screening and clinical samples while the number of VSE and MSSA were obtained from clinical samples only. This practice leads to an underestimation of the real number of VSE and MSSA colonization in contrast to the number of VRE, ESBL and MRSA. When comparing VRE and VSE, there is a higher probability that a patient who has been tested negative for VRE is actually free of the bacteria while there is a lower probability to be free of VSE when the bacteria are not found in clinical samples only. Therefore, the prevention of VSE colonization by probiotics might be easier to achieve than prevention of VRE colonization.

Another limitation of this study is that it is a monocentric retrospective observational analysis. Due to the low number of affected patients and the design of the study, we refrained from inferential statistical analysis. The descriptive results, however, like the finding that the number of *S. aureus* colonized patients remained constant, show convincing evidence of a specific effect on the gut microbiome. Furthermore, it is not very probable that the effects described herein resulted from an unidentified confounding source.

## 5. Conclusions

In summary, the application of probiotics in an early rehabilitation ward to patients treated with antibiotics reduced the percentage of patients colonized with potentially harmful or multi-resistant bowel bacteria (VRE, VSE, and EOEC). That effect was still present after the application of probiotics had stopped. In that setting, bacterial transmission is best explained as a stochastic phenomenon, rather than a linear cause-and-effect event. In the future, such a stochastic transmission model should also be considered when examining cause and effect elements of a transmission chain.

## Figures and Tables

**Table 1:** Number of patients per year on ERWIN colonized with vancomycin-susceptible *Enterococcus faecium* (VSE), vancomycin resistant *E. faecium* (VRE), Gram-negative bacteria producing extended spectrum beta-lactamase (ESBL), methicillin resistant *S. aureus* (MRSA), and methicillin-susceptible *S. aureus* (MSSA). Systematic analysis of genotype mediating vancomycin resistance of VRE was performed from 2013–2016. Ampi sus.: Ampicillin-susceptible.

	2011	2012	2013	2014	2015	2016	2017	2018	2019
*Enterococcus faecium*									
VSE	15	17	32	45	80	66	39	30	15
VSE Ampi sus.	1	0	3	1	6	6	1	0	0
VRE	1	0	2	21	40	21	1	4	2
*van*B VRE			0	17	36	7	1		
ESBL									
*E. coli* ESBL	2	5	2	6	18	14	18	22	15
Other ESBL	5	1	0	4	10	12	3	4	3
*Staphylococcus aureus*									
MSSA	8	12	14	11	11	11	15	15	9
MRSA	2	3	3	5	2	5	5	5	4

**Table 2:** Number of patients per year on wards other than ERWIN colonized with vancomycin-susceptible *Enterococcus faecium* (VSE), vancomycin resistant *E. faecium* (VRE), Gram-negative bacteria producing extended spectrum beta-lactamase (ESBL), methicillin resistant *S. aureus* (MRSA), and methicillin-susceptible *S. aureus* (MSSA). Systematic analysis of genotype mediating vancomycin resistance of VRE was performed from 2013–2016. Ampi sus.: Ampicillin-susceptible.

	2011	2012	2013	2014	2015	2016	2017	2018	2019
*Enterococcus faecium*									
VSE	268	272	346	339	406	401	459	430	367
VSE Ampi sus.	40	44	40	41	48	39	60	54	59
VRE	7	29	21	30	41	34	51	68	98
*van*B VRE			4	21	29	11	17		
ESBL									
*E. coli* ESBL	185	149	153	195	208	203	216	213	193
Other ESBL	56	27	29	40	52	42	48	39	47
*Staphylococcus aureus*									
MSSA	795	791	723	809	775	780	684	716	720
MRSA	167	247	212	96	83	82	55	53	54

**Table 3:** Number of patients per year on ERWIN exhibiting vancomycin-susceptible *E. faecium* (VSE), vancomycin resistant *E. faecium* (VRE), methicillin resistant *S. aureus* (MRSA), and methicillin-susceptible *S. aureus* (MSSA) in blood culture. Gram-negative bacteria producing extended spectrum beta-lactamase (ESBL) and Ampicillin-susceptible *E. faecium* were not isolated from blood cultures.

	2011	2012	2013	2014	2015	2016	2017	2018	2019
*Enterococcus faecium*									
VSE	0	1	0	0	1	0	1	0	0
% of all VSE		5.8			1.3		2.6		
VRE	0	0	0	0	1	0	0	1	0
% of all VRE					2.5			25.0	
*Staphylococcus aureus*									
MSSA	2	4	1	2	2	2	6	2	2
% of all MSSA	25.0	33.3	7.1	18.2	18.2	18.2	40.0	13.3	22.2
MRSA	0	0	0	0	1	1	0	0	1
% of all MRSA					50.0	20.0			25.0
